# Auto-adjusting positive airway pressure: the fine line between engineering and medicine

**DOI:** 10.1007/s11325-025-03409-w

**Published:** 2025-07-28

**Authors:** Ludovico Messineo, David P. White, Bernard Hete, Michael Knepper, Richard Berry, William H. Noah

**Affiliations:** 1 Division of Sleep and Circadian Disorders, Departments of Medicine and Neurology, Brigham & Women’s Hospital & Harvard Medical School, 221 Longwood Avenue, Boston, MA USA; 2 Sleep Centers of Middle Tennessee, Murfreesboro, Tennessee USA; 3 Critical Care & Sleep Medicine, UF Pulmonary, Gainesville, FL USA

**Keywords:** APAP, Continuous positive airway pressure, OSA therapy, Titration, Algorithms, Automatic event detection

## Abstract

Auto-adjusting positive airway pressure (APAP), unlike continuous PAP (CPAP), dynamically adjusts treatment pressure in response to events detected automatically from a derived flow signal. Introduced in the 90’s, APAP quickly became a key tool in sleep clinics, initially serving as a faster alternative to manual titration for patients with obstructive sleep apnea (OSA), and later also as a long-term treatment option to expedite follow-up visits. APAP and CPAP are overall comparable in terms of adherence, efficacy and control of symptoms. However, concern remains that APAP offers less control of chronic health outcomes, such as blood pressure, kidney function and glycemic values. Other APAP-related challenges entail engineering aspects. A major issue is that APAP algorithms—which govern event detection/identification and pressure adjustments—are proprietary of and vary among manufacturers, making them poorly understood by clinicians. Furthermore, APAP algorithms do not always match-up well when compared to both manual titration or manually scored polysomnography, particularly in the presence of unintentional leak. Variability in event detection, leak compensation, and pressure adjustment algorithms among devices adds another layer of complexity to clinical decision-making. All this complicates the management of OSA patients, who could be left with substantial residual disordered breathing, high leak, and a wide pressure range.

This review aims to bridge the gap between the clinical and engineering perspectives of APAP, providing an up-to-date overview of current knowledge and existing challenges that sleep clinicians should consider when managing OSA patients with PAP therapy.

## Introduction

Continuous positive airway pressure (CPAP) has been the gold-standard for treating obstructive sleep apnea (OSA) for over 40 years [1]. While the design of the PAP devices has evolved substantially during this time, their core principles have remained largely the same, i.e., delivering pressurized air through a closed circuit to maintain upper airway patency during sleep. The key components are also mostly unchanged since the early years, and include: (1) a high-speed turbine that generates a range of pressures, typically between 4 and 20 cmH_2_O, (2) a single limb for both inspiration and expiration, (3) an exhaust valve—inserted between the tube and the mask or built-in within the mask—to prevent CO_2_ rebreathing [2, 3].

Two types of CPAP devices are currently available in the market: fixed-level CPAP and auto-adjusting CPAP (or APAP). Fixed-level CPAP—hereafter referred to simply as CPAP—delivers a pre-determined pressure during both inspiration and expiration, with each patient requiring a personalized pressure level that is set to prevent upper airway obstruction, but also to yield the least discomfort and avoid side effects. However, upper airway collapsibility can vary night-to-night with changes in sleep stage, body position, alcohol consumption and nasal obstruction [4]. Over time, this variability is further dictated by changes in BMI [5]. Thus, CPAP may sometimes deliver a pressure that is no longer optimal. APAP was developed early in the 90’s [6] to address this issue. APAP devices are equipped with sophisticated algorithms (proprietary to the manufacturers that produce them [7]) which detect and respond to changes in upper airway inspiratory flow, automatically adjusting the pressure to better meet the patient PAP requirements throughout the night. For example, to properly address respiratory events during rapid-eye movement (REM) sleep, which are typically more severe than during non-REM (NREM) [8, 9], a higher fixed pressure may be needed. In a patient with REM OSA, or where respiratory events coexist in REM and NREM, a manual titration would determine a therapeutic pressure that is adequate for REM, but most likely excessive for NREM. An APAP overcomes this by continuously monitoring upper airway status throughout the night, adjusting pressure accordingly in real time, providing higher PAP in REM and lower in NREM as needed. Additionally, when set to a full range of pressures, APAP automatically determines the average PAP able to resolve all respiratory events, from apneas, to snoring and flow limitation, across all sleep stages and sleep positions for 90–95% of total sleep time (P90 or P95, respectively)—ideally mirroring the outcomes of a manual, in-lab titration [10–12]. However, there is still concern as to whether APAP yields similar outcomes to in-lab titration or to CPAP treatment overall, which will be discussed below.

With the growing prevalence of OSA and obesity [13], both the economic burden of OSA and the clinical ask for titrating studies are expected to rise significantly in the coming years. In this context, APAP is supposed to streamline titrations (if used in place of manual titrations) and potentially decrease follow-up visits (if used chronically), perhaps cutting costs related to clinical resources and personnel. However, without a follow-up polysomnography (PSG) that reliably assesses residual apnea-hypopnea index (AHI) and informs it with oxyhemoglobin saturation and electroencephalogram scoring, patients may not be adequately treated by APAP, whose titration algorithm relies solely on flow-measured AHI. This leaves clinicians relatively in the dark about PAP efficacy and heavily reliant on the device’s algorithm. For example, if the software of the device fails to detect an event, pressure will not increase, and the event will go unrecorded. Conversely, the software may misinterpret variations in flow that are not actual events, such as irregular breathing during wakefulness. As a result, the patient’s upper airway status—and consequently their PAP needs—may be different from what the APAP algorithm assumes.

Adding to this challenge, there are few regulations or guidelines governing how manufacturers bring new devices or algorithms to the market [14]. This leads to quite a few different devices with potentially different branding terms for analogous concepts [15], arguably creating confusion for clinicians. Worse, since these algorithms are proprietary and never disclosed, their functioning remains largely opaque. This lack of transparency impacts not just the science, as research into these algorithms is severely limited, but also patients, given the absence of robust real-world testing before these technologies are released for clinical use [16]. Clinicians are also affected, as they often lack clarity on how the device determines its treatment adjustments, which may lead to selecting a device that is not optimal for a given patient. Although algorithm outputs often present flow shapes and event flags, interpreting these can be challenging and time-consuming, and the APAP summary report most frequently becomes the only part of the output that is looked at during an outpatient visit.

Despite substantial research, the effect of APAP on a variety of OSA outcomes is not completely clear, due to different results from numerous trials comparing its effectiveness vs. CPAP. In addition, data clearly showing how effectively APAP algorithms work are limited, with study results that are often inconsistent or not easily grasped by physicians. A comprehensive synthesis of existing evidence, integrating both updated clinical outcomes and technical aspects of device functioning to help sleep clinicians navigate the APAP black box, is currently missing from the medical literature. To address this, we assessed major online databases (PubMed, Google Scholar, Harvard Library) from their inception to the current date, and conducted a narrative review with the goal of summarizing what is known and highlighting existing gaps in medical and engineering knowledge to help strategize future research and development of PAP technologies in a way that is more accessible to the sleep community.

## APAP VS. CPAP: cost-effectiveness, pressure settings and treatment outcomes

Shortly after its introduction and throughout the 2000s, the use of APAP progressively gained traction as a faster alternative to manual titration. Clinicians saw APAP as a way to titrate more patients and save on lab resources. The standard APAP titration approach involves using the device overnight for 1 to 7 days at home to determine P90 or P95, then switching to CPAP with a fixed pressure set to that value. Additional advantages over in-lab titration included reduced time to therapy initiation, improved access to care, and greater patient preference, as most patients intuitively prefer spending the night at home rather than in the lab [17].

There is general consensus that APAP titration addresses respiratory events similarly to in-lab titrations [17–23], but provides, on average, 1–2 cmH_2_O higher pressures [24–27]. However, this notion has been challenged in the literature [23, 28]. Regardless, as aforementioned, frequent follow-up visits are still required for the patient on CPAP to ensure that the titrated pressure remains appropriate for their needs, as the minimal effective pressure can change overtime [5, 29].

Currently, most patients are given an APAP device for both titration and treatment straight after OSA diagnosis. In a pre-pandemic study including ⁓2.6 million OSA patients, 50% of the prescribed devices were APAP, 41% were CPAP, and the remaining 9% were bilevel PAP or adaptive servo-ventilators [30]. Since the pandemic encouraged home-diagnosis and follow-ups, it is not unfair to assume that APAP prescription surged even more in the past 2–3 years. According to industry sources, over 90% of devices used for home therapy in the United States are now APAP. Notably, this does not mean that > 90% of patients are actively using APAP mode, as most APAP devices can also be set to deliver fixed-pressure PAP. Empirical data also say that the majority (⁓65%) of the patients who are given an APAP for treatment are left with a full pressure range (4–20 cmH_2_O) for the duration of their therapy.

There are several advantages of using APAP over CPAP. The overall process from titration to treatment initiation is likely cheaper when APAP is used [31–34]. A study found that APAP titration was about 16% cheaper than manual, in-lab titration [20]. In another study, home sleep apnea testing plus APAP titration was 25% cheaper than the corresponding in-lab procedure [34]. By contrast, the APAP cost/benefit ratio in the long term is not completely clear as medical costs vary between countries and new PAP machines are continuously introduced into the market. Although data suggests that APAP has an edge on CPAP for titrations, CPAP may be better in the long run. A Swiss study reported that costs after 2 years of follow-up were similar for CPAP and APAP [35]. Conversely, in a Portuguese medical setting and in patients optimally adherent to APAP with no reported leak and an automatic event detection (AED) AHI of less than 5 event/h, a transition from APAP to CPAP reduced costs by €120/patient in a two-year period [36].

Nevertheless, the main presumed advantage of chronic use of APAP over CPAP was improved overall treatment adherence, which has long been the primary limitation of PAP therapy [37]. This belief was based on several reasonable assumptions. First, APAP reduces mean pressure vs. CPAP by roughly 10–40%, due to decreased pressure needs during both non-supine and NREM sleep [38–52], despite increased peak pressure [45]. Second, there is some evidence suggesting reduced PAP-associated side effects/complaints with APAP [38, 53], possibly driven by the lower mean pressure. APAP improvements included reduced machine noise [51, 53], less perceived discomfort from reduced expiratory pressure [38, 51, 53, 54], better subjective sleep quality [54], and less bloating/aerophagia [40]. Decreased nasal symptoms were also reported in some [38], but not all studies [55]. However, it has to be noted that several other studies have shown no difference in side effects between CPAP and APAP [18, 35, 40, 49, 53, 56], with additional evidence of reduced comfort with APAP possibly due to the oscillating pressure [52]. Third, reduced leak was observed with APAP in some studies [38, 45, 57], but not all [39, 41, 43, 44]. Since peak pressure is a key contributor to unintentional leak [58], it is possible that leak increases with APAP, especially when the devices are set with a wide pressure range. To this point, a randomized controlled trial found that therapeutic pressure and unintentional leak were higher after a week of APAP (set to 4–12 cmH_2_O) vs. CPAP (set up using a prediction formula [59] with PAP adjustments allowed via telemonitoring) [60].

**Fig. 1:**
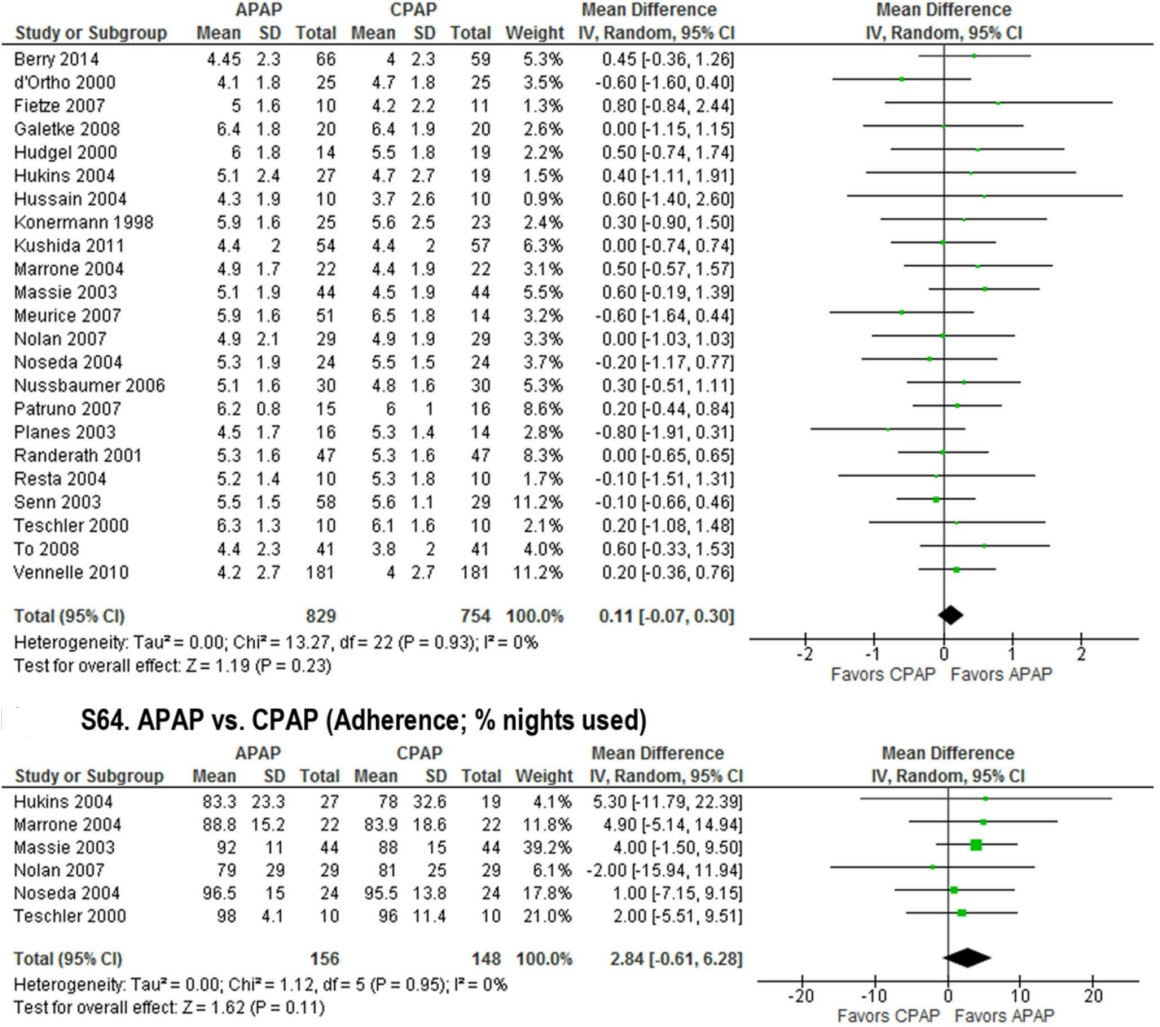
A meta-analysis of 23 randomized controlled trials demonstrated no clinically significant difference in average hours of use in adults with obstructive sleep apnea treated with auto-titrating positive airway pressure (APAP) compared to continuous PAP (CPAP). In addition, a meta-analysis of 6 of these studies demonstrated no clinically significant difference in percent of nights PAP was used. Reproduced with permission from Patil et al. [18]

Despite the above and some early data suggesting greater APAP usage time [46–48, 54], most studies observed adherence on APAP to be similar to that of CPAP [23, 38, 41, 43–45, 49, 51–53, 55, 61–67] (Fig. 1). Increases in adherence on APAP vs. CPAP were absent also when examining only selected people with reported complaints of aerophagia [40]. Some authors even postulated that high pressure on fixed CPAP is a criterion for better adherence [68].

Multiple studies have observed that the APAP-driven improvements in excessive daytime sleepiness measured via either the Epworth Sleepiness Scale or other tests (e.g., Osler, multiple wakefulness test) [23, 27, 38, 40, 41, 43, 44, 46, 47, 49, 51, 53–56, 59, 62–67, 69], in quality of life [38, 40, 41, 43, 51, 53, 55, 59, 65–67] and in the AHI [23, 38–41, 43–47, 49, 51, 52, 54–56, 59, 62–67, 69–71] were similar to those seen with CPAP. A similar equal effect was also reported with selected patient groups, such as those with high night-to-night variability in pressure requirements [72], or mild-to-moderate OSA [57]. The absence of clinically meaningful outcome differences between treatments [35, 73, 74] was also confirmed after long follow-ups (2 years) [35] and with repeated meta-analyses [18, 42, 50, 75–77]. Mixed results for subjective preference were also reported [49–51, 53].

Based on the above, a task force recommended no preference between APAP and CPAP for OSA patients without important comorbidities [12, 18], while an in-lab, manual titration should always be the first choice when other diseases potentially influencing respiration during sleep are present (e.g., heart failure, chronic obstructive pulmonary disease, obesity hypoventilation syndrome, etc.) [12].

**Fig. 2:**
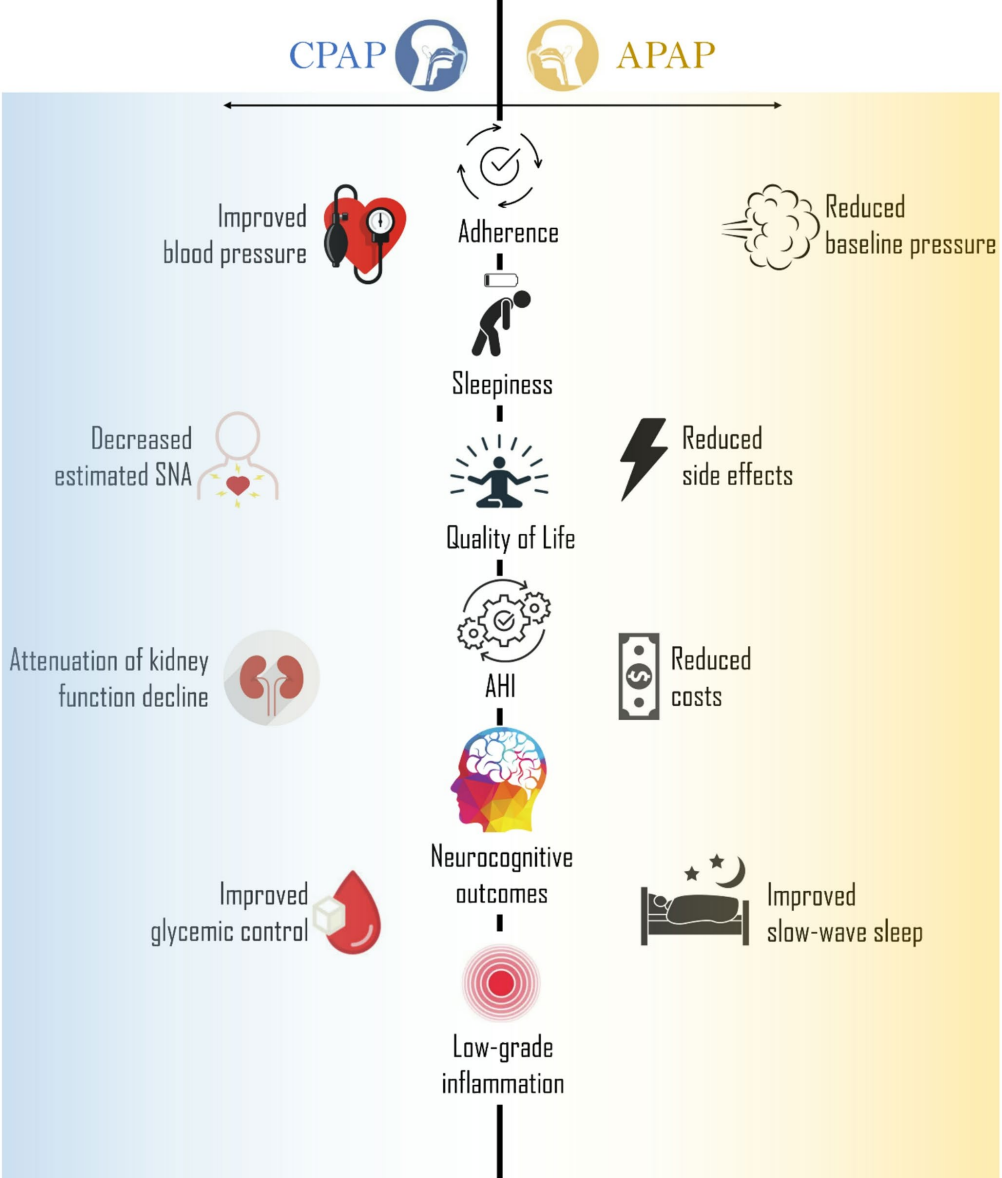
Summary illustration comparing the effects of auto-adjusting positive airway pressure (APAP) and continuous positive airway pressure (CPAP) on key treatment and health outcomes. The central axis represents outcomes with no significant difference between the two treatments, supported by reasonable evidence. Advantages specific to APAP and CPAP are displayed on either side, with greater distance from the center indicating a stronger effect or (estimated) higher level of supporting evidence

**Fig. 3:**
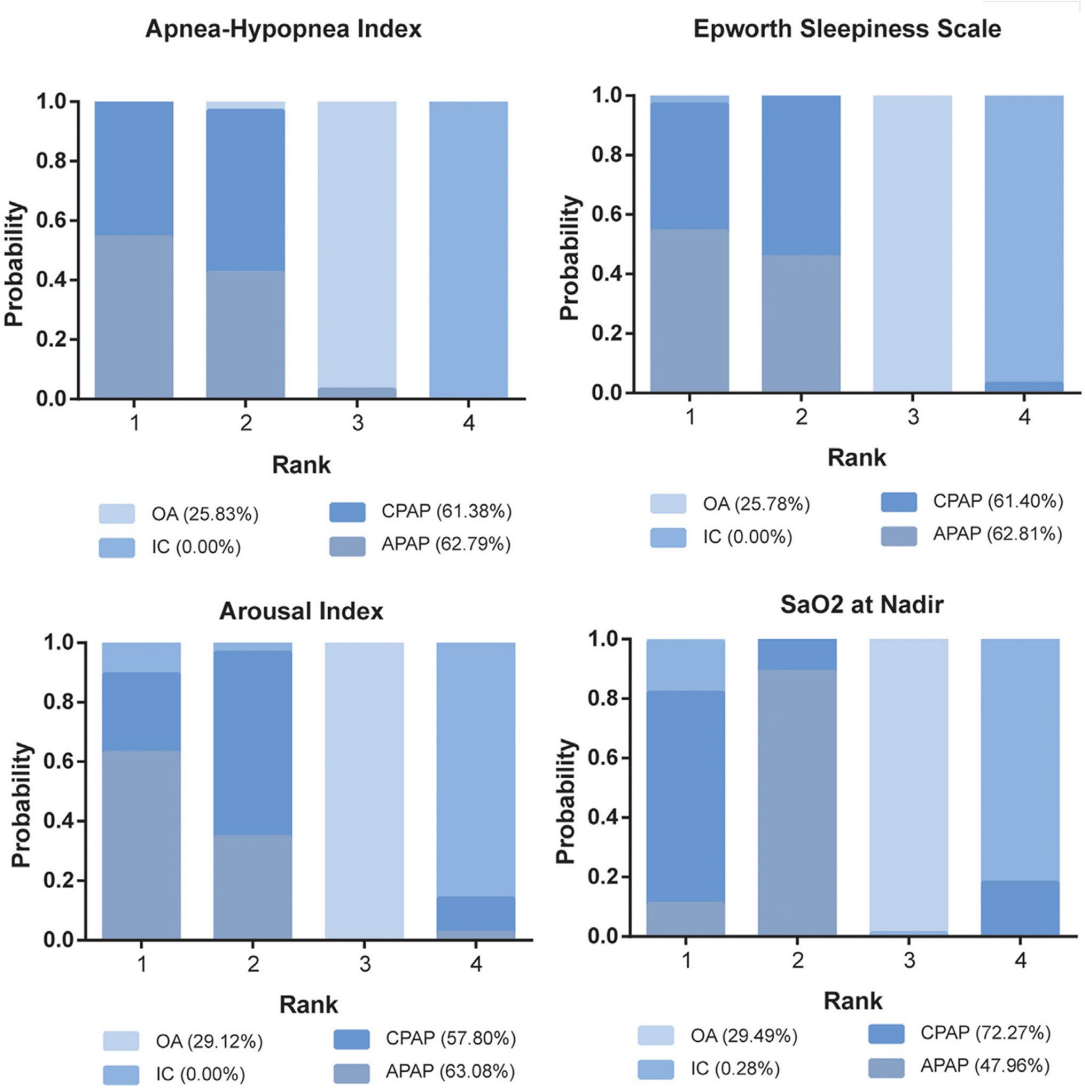
Stacked bar charts showing the rankings of four therapies for 24 h systolic blood pressure (SBP), 24 h diastolic blood pressure, daytime SBP, daytime SBP, nighttime SBP, and nighttime SBP according to a network meta-analysis. This ranking analysis was based on the surface of cumulative ranking curve area (SUCRA), which estimates the probability of a treatment being the “best” option among those compared. Treatments in Rank 1 are potentially more favorable than those in Rank 2, etc. Higher cumulative SUCRA values (percentages in brackets) indicate a stronger overall ranking by incorporating probabilities across all ranks. For example, even though OA and IC never rank first in the plot for daytime SBP, their probabilities of ranking in other positions contribute to their likelihood of being the best treatment in 13.63% and 23.73% of cases, respectively. Abbreviations: CPAP, continuous positive airway pressure; APAP, auto-adjusting positive airway pressure; IC, inactive control; OA, oral appliance. Reproduced with permission from Liu et al. [77]

Since APAP and CPAP are similar modalities, it has generally been assumed that APAP provides comparable protection against adverse health outcomes as CPAP. However, this is still controversial, as differences between the two treatment modalities have been reported (Fig. 2). Although numerous studies and meta-analyses assessing blood pressure as a secondary outcome reported no significant difference between APAP and CPAP, two studies specifically designed to compare between-treatment differences in health outcomes found that CPAP led to greater blood pressure reductions at both three [78] or four [79] months. A network meta-analysis ranked CPAP above APAP in probabilities of reducing daytime and night-time blood pressure (Fig. 3) [77]. A potential explanation for the reduced effect of APAP on blood pressure came from the observation that sympathetic output—as per heart rate variability [80, 81] or arterial stiffness measured via pulse wave amplitude [82]—decreases less on APAP vs. CPAP. This may be due to undertreatment of OSA with APAP, as some algorithms yielded only partial control of respiratory events (see below) [83]. However, it is important to keep in mind that the effect of PAP on any type on blood pressure is modest [84, 85].

APAP seems to have little effect on glycemic control [86, 87], while, on CPAP, a better glycemic profile, especially overnight, was reported [88]. The relevance of this difference is supported by a study proposing a decreased homeostatic model assessment of insulin resistance (HOMA-IR) on CPAP at 3 months—which indicates improved insulin sensitivity—, but not on APAP [78]. However, the evidence of a positive effect of CPAP on glycemic control is quite inconsistent [18].

In addition, in a large cohort, it was demonstrated that APAP did not attenuate the progressive decline in kidney function observed in patients with chronic kidney disease as effectively as CPAP [89]. This was hypothesized to be linked to a poorer hemodynamic profile on APAP vs. CPAP [90].

Some authors have proposed that, in patients requiring frequent pressure adjustments throughout the night, APAP, may decrease sleep efficiency [91], although this remains very speculative [92]. APAP algorithms which mistakenly over-titrate might also lead to treatment-emergent central sleep apnea (TECSA) through tidal volume augmentation [93].

In contrast, one meta-analysis suggested that APAP led to increased slow-wave sleep vs. CPAP [42].

No differences in neurocognitive outcomes [41, 66] or low-grade inflammation [35, 78] have been reported to date between treatment modes.

Finally, while there is concern that PAP might be associated with adverse health outcomes [94], it remains uncertain whether APAP differs from CPAP in this respect, as both have been linked to unfavorable effects on pulmonary vascular inflammation markers [95–97]. In summary, APAP and CPAP seem equally effective in titrating to therapeutic pressure and treating OSA in the long-term, although APAP did not yield improvements in PAP adherence, which remains low. Differences in costs and chronic management of adverse outcomes are yet to be fully clarified, but most evidence leans towards better outcomes with CPAP. Further supporting this notion, transitioning patients with side effects or poor adherence from APAP to CPAP yielded improvements in daytime sleepiness and adherence, particularly in those with higher baseline arousal indices and lower nadir saturation [98]. Preliminary evidence also suggests that most moderate-to-severe OSA patients could be fully treated with no pressure titration by going directly to fixed CPAP of 10 cmH_2_O, without significant changes in subjective preferences, objective sleep quality or breathing effort vs. lower PAPs [99]. Notably, effort and breathing difficulty were reported to onset from 12 cmH_2_O [99].

It is important to keep in mind that many of the reported inconsistencies between studies might be due to differences in the APAP algorithms used, which are constantly evolving and now differ from those of the past (i.e., studies performed in different decades might not be comparable). Given the widespread use of APAP, a full understanding of these algorithms seems crucial to ensuring APAP is prescribed appropriately and tailored to patient needs.

## Differences in automated event detection (AED) and leak estimation algorithms, and the risk of residual OSA

**Fig. 4:**
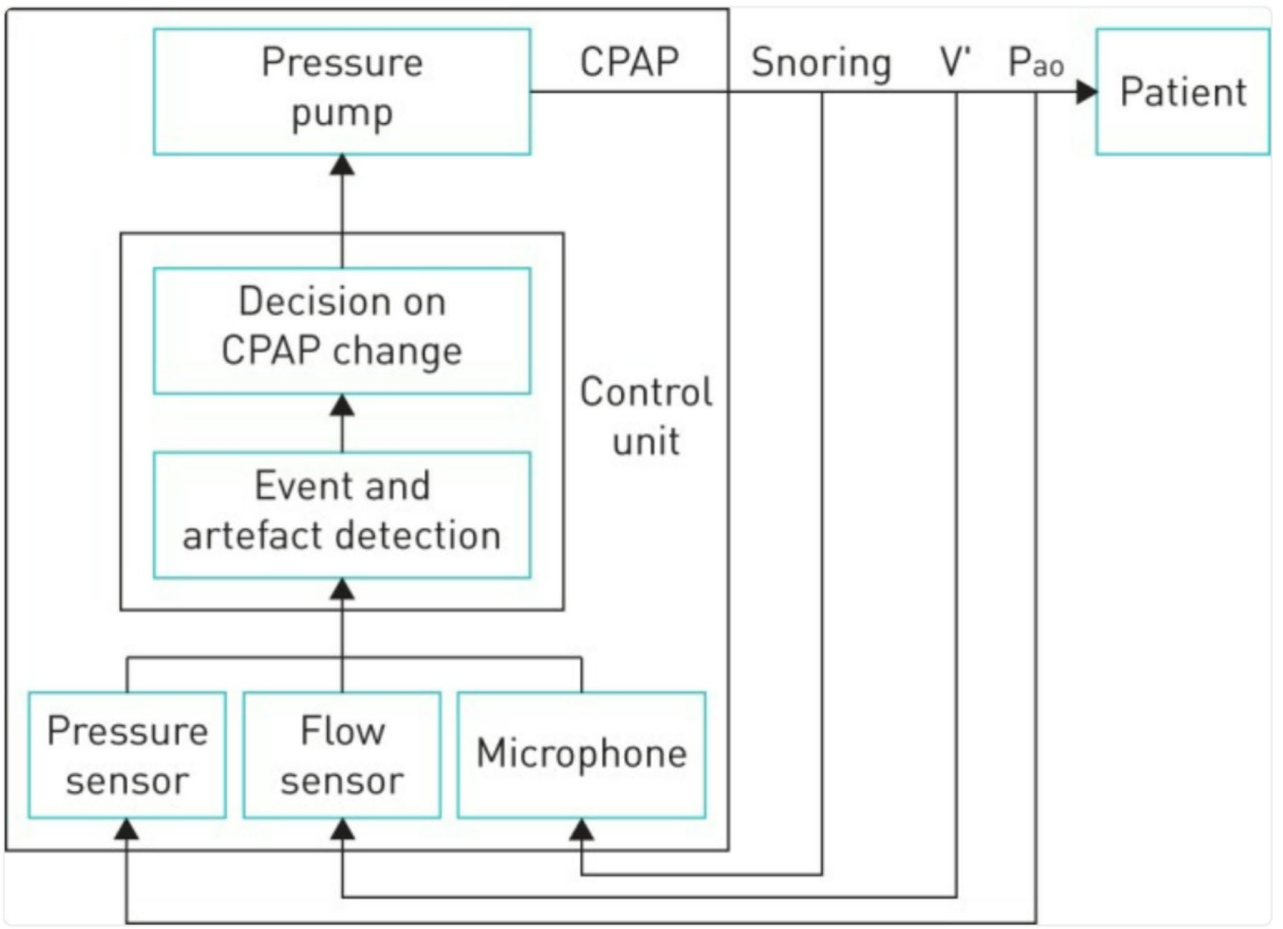
Schematic diagram illustrating the functional principles of a device for applying automatic continuous positive airway pressure (CPAP). V′ and P_ao_ are flow and pressure measured at the patient’s airway opening. Reproduced with permission from Farrè et al. [14]

**Fig. 5:**
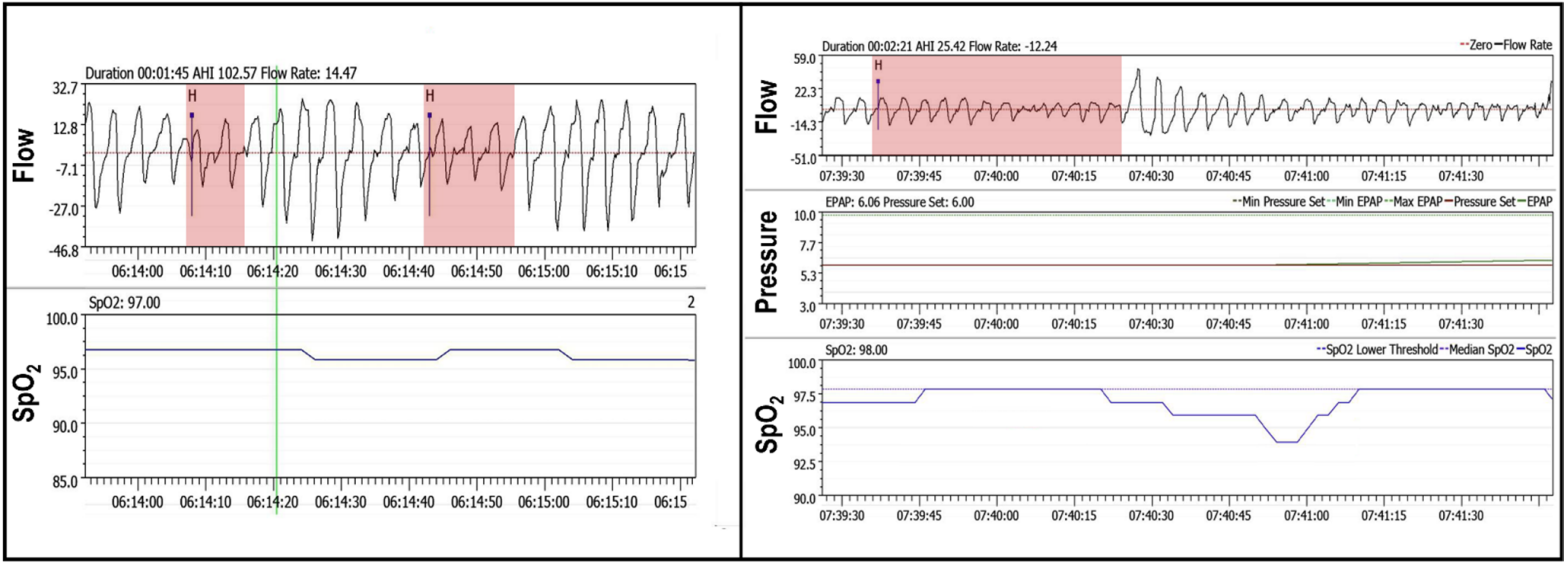
Real-world example traces from an APAP device. Flow and pressure signals were synchronized with the oxyhemoglobin saturation (SpO_2_) signal using a specialized software. The left panel illustrates two hypopneas flagged by the device (red shading), yet neither is associated with a desaturation, making them unlikely to be true events. By contrast, in the right panel, a flagged hypopnea lines up correctly with a desaturation (i.e., a true event was detected). Integrating SpO_2_ alongside the estimated flow signal from the device would enhance event detection accuracy

APAP algorithms are designed to detect, in real time, apneas, hypopneas, and flow limitations, and differentiate between actual respiratory events and non-respiratory disruptions like coughing, swallowing, or mouth breathing. Additionally, the algorithms assess and quantify unintentional leak. Finally, they decide whether the pressure should change in response to automatically detected events and/or in the presence of unintentional leak. Inputs to the algorithms are measures of flow, pressure and a snoring detector (e.g., through vibrations in pressure). These signals are fed into a control unit that determines the corresponding pressure changes (Fig. 4). This dynamic adjustment is more challenging than simply scoring events in a retrospective session as it is based on a single signal (estimated patient flow) rather than multiple ones (flow and oxygen saturation, with the possibility of adding other supporting signals) used in manual scoring, and it must respond instantly to any sensed breathing irregularity. Given these challenges, APAP algorithms have room for error (Fig. 5). Thus, due to the proprietary nature of the algorithms, which limits available information, it is key that sleep clinicians are aware of the limitations of these devices and understand as much as possible how algorithms detect and classify events.

Different algorithms have different ways to quantify the flow signal (the algorithm foundation for event detection), but generally they filter the signal to distinguish between patient flow and leak. High-pass filtering removes offsets and isolates patient flow by eliminating leak, while low-pass filtering removes high-frequency content, such as breathing, to estimate leak. These filtering processes also help smooth out variability, i.e., reduce the impact of low-frequency (e.g., sighs, swallows, or brief flow interruptions such as irregular breathing activity during wake time) and high-frequency (e.g., cardiogenic artifacts) fluctuations in flow. The filtered signal is then used to determine an average value over a set period for further analysis. Once the signal is processed, the device evaluates changes in flow over different time windows to determine whether an event has occurred [100, 101]. Each manufacturer’s algorithm undertakes this procedure differently, which may eventually result in quite different identification of events and pressure adjustments.

**Fig. 6:**
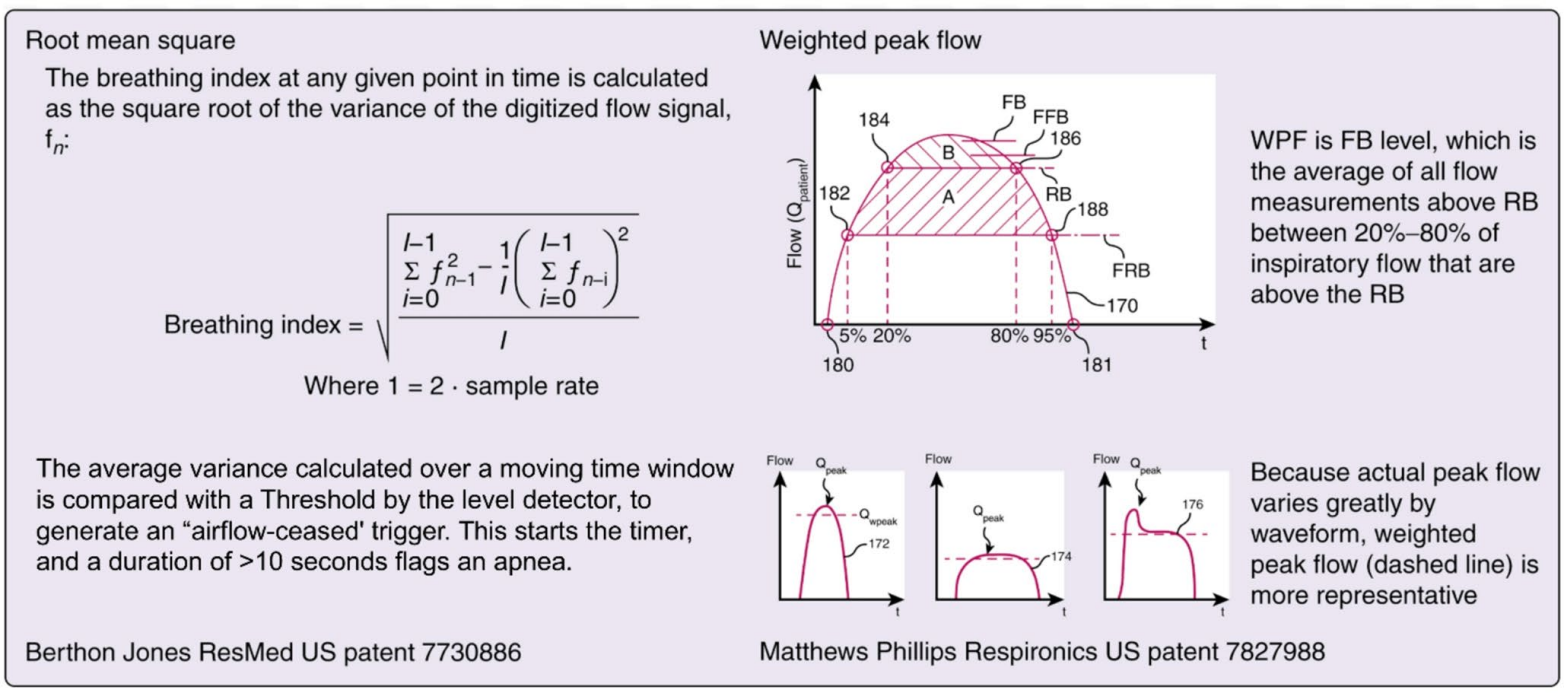
Signal processing methods. 180 and 181 refer to the start and the stop point detected by the algorithm for the flow curve analysis. 182, 184, 186 and 188 refer to the illustrated percentage in inspiratory volume. Flatness round baseline (FRB) is determined to find if there are any points between 5% and 95% of the flow curve that are below the 5% or the 95% of the flow value on the y axis. In practice, this is done to understand if there is any flow point in the middle of the flow curve that is below a line connecting the flow points at 5% and 95% of the curve. Roundness baseline (RB) is determined similarly, but using 20% and 80% of the flow curve. Flatness flat baseline (FFB) and Flatness baseline (FB) are calculated as the average of all flow points above FRB and RB, respectively. Hence, FB corresponds to weighted peak flow. The striped areas correspond to volume measures: the volume corresponding to FFB is A + B, while the volume corresponding to FB is B. Flow patterns 172, 174 and 176 are exemplified to illustrate that peak flow (Q_peak_) does not always correspond to weighted peak flow (Q_wpeak_; dashed lines). FRB, RB and FFB are used to control respiratory flow limitation. Modified and reproduced with permission from Freedman and Johnson [100]

For example, Philips Respironics uses a method called weighted peak flow (WPF) to estimate flow, which first identifies the inspiratory period in the flow curve and then calculates the average inspiratory volume as the mean of the points falling between 20% and 80% of the flow curve (Fig. 6). The actual WPF is then compared to the average WPF of a previous 4 min moving window (baseline) to determine if a reduction in flow occurred [102, 103]. If this ratio drops below 20% for at least 10 s, the device flags an apnea (event termination is marked when a breath exceeds 30% of the baseline). If, instead, it drops by 40–80% for 10 s (ending when current WPF returns above 75% of baseline values), a hypopnea is scored.

By contrast, ResMed uses a method known as root mean square of the variance of the flow to measure flow changes from breath to breath. Instead of looking at individual flow points in isolation, the algorithm calculates an average airflow over a set period and tracks deviations from that average, producing a continuous output called the breathing index (Fig. 6). These deviations are averaged and then square-rooted to reduce the impact of outliers. To detect apneas, the algorithm compares short-term (e.g., a breath or 2 s) to long-term windows (e.g., 5 min, the baseline) of analyzed flow variance and flags any reduction of the breathing index below 25% (for 10 s; below 50% for hypopneas) of the baseline reference [104].

Similarly, SleepRes devices [105], use an algorithm that monitors the time-course of the ratio of short- to long-term statistical variance in flow (algebraically captured in a variable called *R*) to recognize events. Based on the level and duration of reduction in *R* (from least to most), events are scored with progressive severity (mild, severe hypopneas, apneas). This approach yielded similar results to Philips Respironics’s AED in a recent, preliminary investigation (Table 1).

**Table 1:** Comparison of OSA severity detection between the APAP algorithms of Philips respironics and sleepres

Characteristic	PR APAP	SleepRes APAP
	*Estimate*	*Adjusted mean difference* *[95%CI]* *P-value*
AHI_4_ (events/h)	2.6	−0.2 [−0.8, 0.4] 0.451
AHI_3a_ (events/h)	7.9	−0.4 [−1.9, 1.4] 0.669
NREM AHI_4_ (events/h)	2.1	−0.1 [−0.7, 0.5] 0.614
REM AHI_4_ (events/h)	4.6	−1.5 [−2.7, −0.0] 0.046
NREM AHI_3a_ (events/hour)	7.1	−0.4 [−1.9, 1.2] 0.597
REM AHI_3a_ (events/hour)	10.9	−0.6 [−3.3, 2.4] 0.661
AHI_4_ supine (events/h)	3.4	−0.2 [−1.2, 0.9] 0.663
AHI_3a_ supine (events/h)	9.7	−0.9 [−3.1, 1.6] 0.448

All AHI values were square-root transformed for analysis and back transformed for presentation. Models were adjusted for period, sequence, baseline AHI and percent of time spent sleeping supine in the corresponding sleep stage. Sensitivity analyses with further adjustment for body mass index, age and race did not meaningfully affect the examined variables. Thirty-two participants were included in the analysis according to a modified intention to treat approach (i.e., if they performed at least one treatment visit). These data refer to a yet unpublished clinical trial which aimed to assess the effect of the SleepRes APAP vs. Philips Respironics APAP. PR, Philips Respironics; APAP, auto-adjusting positive airway pressure; AHI_3a_, apnea-hypopnea index where hypopneas are scored if associated with 3% oxygen desaturation or cortical arousal; AHI_4_, apnea-hypopnea index where hypopneas are scored when associated with 4% desaturation; REM, rapid-eye movements; NREM, non REM

**Fig. 7:**
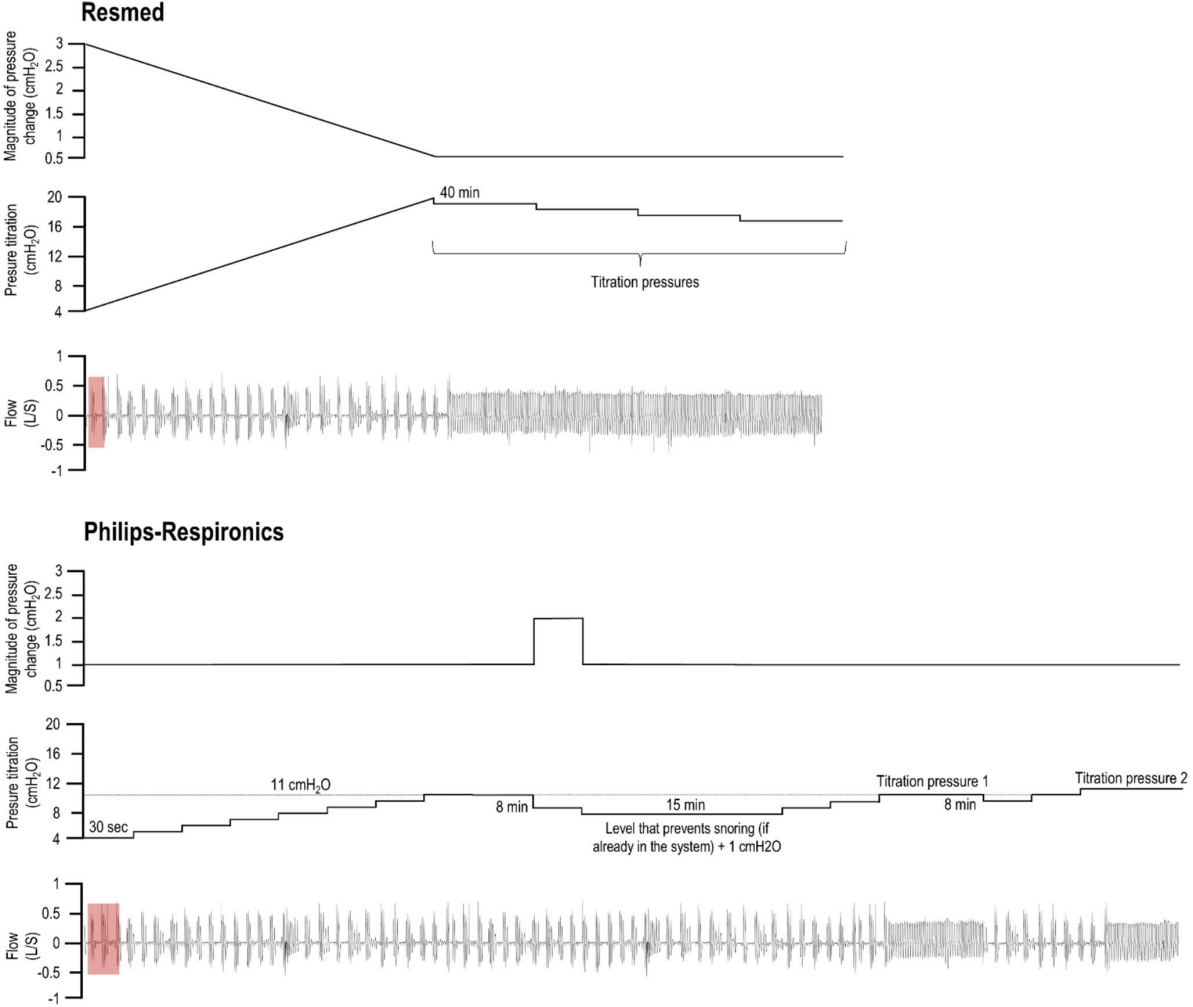
Example flow and pressure traces to illustrate the main principles of the titration algorithms of two manufacturers [103, 104]. Note that time is not represented in scale and the figure assumes all illustrated events are obstructive, with no concurrent leak. Titration pressure is indicated when stable breathing occurs. Different levels of titration pressure contribute to the determination of P90/P95. In the top panel, Resmed’s algorithm is described. A single respiratory event triggers a pressure increase. Pressure is raised more if events occur at low vs. high baseline PAPs. The rate of rise in pressure is limited to 12 cmH_2_O per min. Pressure is decreased by 1 cmH_2_O for each 40 min of apnea-free breathing (or 20 min, if no flow limitation is detected). Philips-Respironics’s algorithm is presented in the bottom panel. Every two respiratory events trigger a 1 cmH_2_O increase in pressure in a “titration cycle”, with a ceiling at 11 cmH_2_O, where pressure is held for 8 min. If events persist, pressure is first reduced by 2 cmH_2_O and then further reduced to the pressure that prevents snoring (if such pressure was already determined by the algorithm previously) + 1 cmH_2_O for 15 min. At the end of this period, all titration limits are cleared and a new “titration cycle” can start, if events keep occurring. If, during the 8 min hold, there is no events, the 11 cmH_2_O ceiling is cleared and a new “titration cycle” can begin, if deemed necessary (above 11 cmH_2_O, the maximum allowable pressure increase for the “titration cycle” is 3 cmH_2_O), otherwise pressure can be reduced by 1 cmH_2_O every 8 min. Notably, the starting pressure is 4 cmH_2_O to avoid circuit rebreathing [2, 3]

Increments in PAP are delivered based on flow reductions, and each machine has its own way to respond to alterations in flow (Fig. 7). With all manufacturers, the general rule is that, if one or two events are detected within a set timeframe, the device increases pressure by 0.5-3 cmH_2_O (the amount generally depends on the level of the background pressure), while, if no events occur, the pressure is gradually reduced [102]. Notably, pressure adjustment algorithms are featured in all APAP devices, while AED algorithms are in both APAP and CPAP devices.

But how well does this AED algorithm perform? Initially, devices had different efficacy in identifying events, flow limitation [106, 107], and snoring [106, 108]. Algorithms improved with the years [23, 109–112], and nowadays, devices seem to largely perform similarly to manual PSG scoring. Indeed, research has shown that the gold-standard AHI (i.e., from the manually scored PSG) and the AHI from the estimated flow signal are similar [28]. However, the latter was demonstrated to being a more inconsistent metric than the former, as the difference between the two (AHI_PSG_ – AHI_FLOW_) has wide limits of agreement, indicating considerable variability for AED-determined AHI [113, 114]. A study showed that the optimal cutoff for the AED-AHI to identify residual OSA (defined as PSG-derived AHI equal or above 10 events/h) was 8 events/h, with a sensitivity of 94% and a specificity of 90% [109]. Slightly lower values were reported in another study when an AED-AHI above 4 was used to identify a manually-scored AHI from PSG equal or above 10 [115]. Gagnadoux and coworkers showed good accuracy between manual and AED scoring, but patients with difficult CPAP titrations (those who likely require the most precise event detection) were excluded [116]. In contrast, another work demonstrated that AHI_FLOW_ could detect PSG-scored events (however scored with the 4% desaturation criterion) with just over 50% accuracy, due to a high number of false positive (however, 98% specificity was reported) [117]. More inconsistent association between manually and automatically scored events was described in other studies [113, 118, 119], even when manual scoring was done using the raw flow signal from the device (and these discrepancies did not improve overtime) [118].

The performance of the AED algorithm can be affected by unintentional leak [120]. The degree by which leak impacts event detection depends mostly on the device brand, both historically [121] or in more recent models [122]. Different manufacturers use different methods for calculating and reporting leak [123], and sometimes these calculations may be confounded by the randomness [124] or the discontinuity of the leak [125], which could then affect the output parameters (e.g., tidal volume, AHI, pressure) [122, 126]. Generally speaking, all devices try to compensate for excessive leak by altering the turbine speed when unexpectedly large deviations in flow are encountered. For example, with a large leak (above twice the expected intentional leak for Philips-Respironics devices or > 24 L/min for ResMed devices), the device may try to compensate by reducing PAP. However, this could lead to under-titration at times. In other cases, expiratory mouth leak could be interpreted by the algorithm as persistently increased airway resistance or ineffective pressure delivery, and thus lead to over-titration. Given these potential problems, leak can increase pressure fluctuations and eventually trigger arousals, which elevate flow and propagate further pressure swings [91]. This can also drive carbon dioxide below the apnea threshold and yield ventilatory instability, including period breathing [119], and contribute to sleep fragmentation [127] or TECSA onset [93]. On APAP, excessive leak can originate from a high baseline pressures [58], but might also be associated with external factors such as wearing nasal pillow masks, male sex, aging, and increased abdominal fat [128].

Understanding the limitations of AED-scoring for the follow-up of residual OSA is crucial. In fact, residual events while on PAP is a relatively frequent occurrence overall [55], and may be due to unresponsive anatomical factors, lower PAP tolerance [18], or severely abnormal physiological factors such as high loop gain or low arousal threshold [129, 130]. However, misclassification of events by the device may also play a role [131]. Another potential cause of persistent OSA during APAP treatment is inadequate pressure adjustments (based on poor recognition of events or on insufficient titration in response to events, especially hypopneas, see below). Although the findings described in the previous section suggest that APAP and manual titrations are equivalent, some studies have reported that over 40% of patients may not be optimally treated on APAP [83, 132, 133]. Such failures may result from high leak, a history of cardiac disease, co-existing central apneas or a high arousal index [132].

In summary, different algorithms, varying by manufacturer, are used in AED. While APAP and CPAP algorithms perform overall well, research suggests that the AHI_FLOW_ is not equivalent to manual scoring, especially in the presence of unintentional leak. Thus, the leak output in the device report should always be carefully reviewed, keeping in mind that automatic leak detection reflects only 30% of the leak episodes identified by the PSG, with the Philips Respironics algorithm appearing less accurate than ResMed’s [127]. Accordingly, subjective complaints indicative of potential leak (e.g., exaggerate noise from the device, dry eyes, air blowing on the bed partner, etc.) should always be investigated.

Overall, when the leak is negligible and the AED-AHI is below 10 events/h, the patient is likely well treated [110]. This is particularly true when the AHI reduction is accompanied by the resolution of night-time and daytime symptoms.

When severe residual OSA occurs during APAP therapy, a manual titration should be strongly considered. This is also the case when a large leak is suspected, when subjective sleepiness does not resolve despite adequate treatment adherence, or when there is a substantial change in BMI or the onset of a new comorbidity.

## Variability in event type (obstructive versus central) detection across devices

Conflicting evidence suggests that APAP devices may [134] or may not [83, 120, 135] perform similarly across different models. Potential differences may arise from how each device accounts for leak (see above) [120], from over-detection of events during wake [136], but also from different abilities in detecting event types [137, 138]. Hypopneas scored by the device, for example, have been reported to be up to twice as frequent as those scored manually [139]. This discrepancy may get even worse in the presence of a leak [122, 139–143]. Between-device differences in flow limitation detection have also been observed [144]. Most importantly, some algorithms have difficulty discriminating central from obstructive events [142]. Li and coworkers reported that only 62% of true central apneas were recognized by AHI_FLOW_ as such, while only 72% of actual obstructive events were correctly classified by the algorithm as obstructive apneas/hypopneas [137]. The intraclass correlation coefficient between manual scoring of obstructive apneas plus hypopneas and AED scoring was very low in one study [115]. This is important in the context of titrating the pressure, as the device will not raise the pressure in response to a central event, when the upper airway is not obstructed. Thus, if event types get misclassified, the device could over-titrate (if it recognizes an excess of obstructive vs. central events) or under-titrate (if the opposite happens).

Each manufacturer uses its own method to identify event types. When absence of flow is detected, Philips Respironics’s and SleepRes’s algorithms provide pressure pulses that induce (if the airway is open) or not (if the airway is obstructed) corresponding flow pulses, respectively flagging central or obstructive apneas. The main drawback of this method is that, when reviewing the waveform output from the device, the presence of multiple pulses within an event can make it challenging to determine which events are true apneas.

**Fig. 8:**
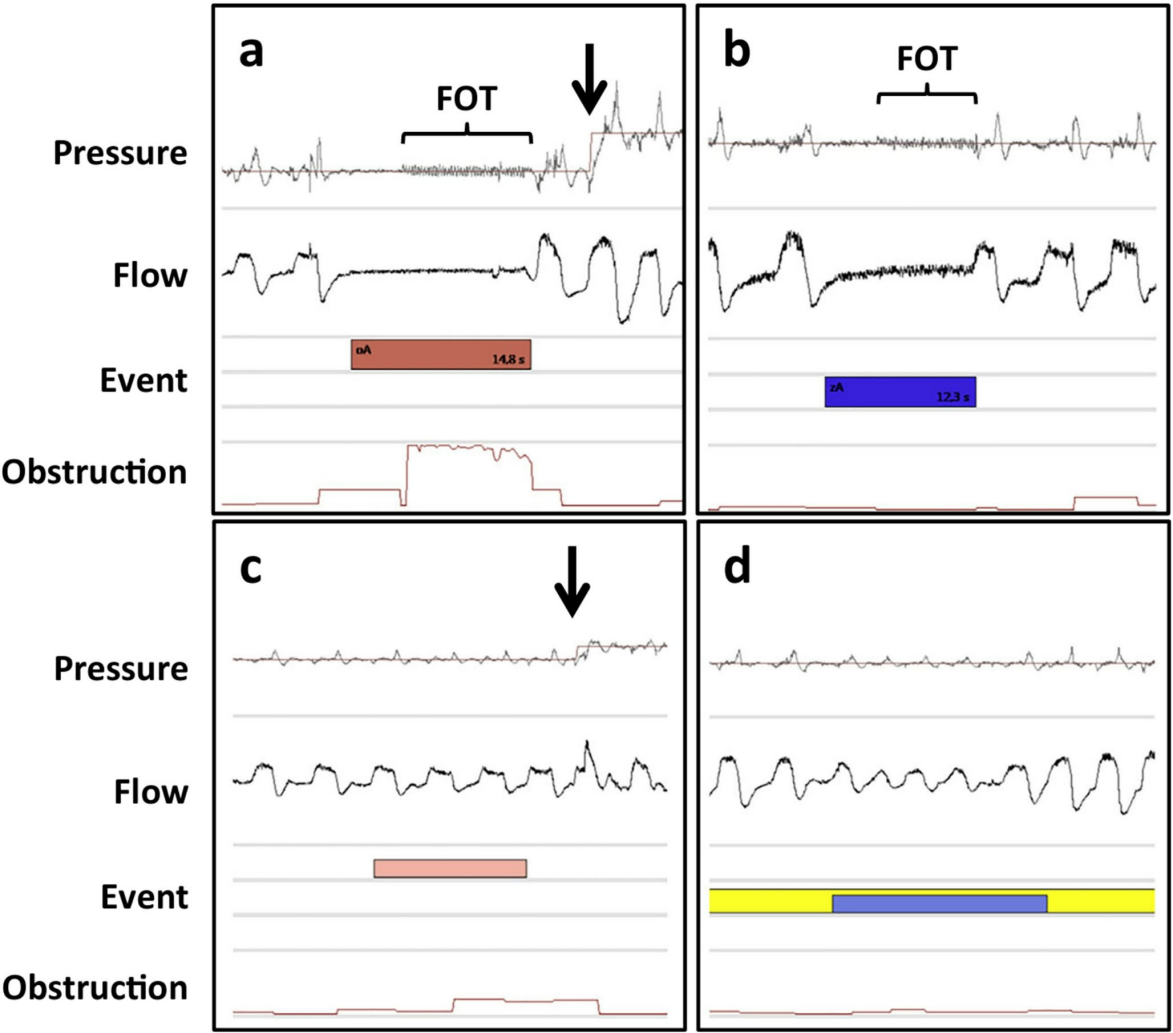
Exemplary sections from device data showing an obstructive apnea (**A**), and a central apnea (**B**), identified through forced oscillation technique (FOT). An obstructive hypopnea (**C**), and a central hypopnea (**D**) were detected through flow contour analysis (e.g., flow flattening examination). The arrow marks the point of pressure increase. Reproduced with permission from Herkenrath et al. [148]

Conversely, Resmed uses the forced oscillation technique (FOT), a technology introduced in the ‘50s that generates sinusoidal pressure oscillations after about 4 seconds of no airflow, to measure resistance in the upper airway [145, 146]. Similarly to above, if the pressure oscillations superimpose on the flow signal (i.e., open airway), the event will be flagged as central. Otherwise, it will be flagged as obstructive [63, 147, 148] (Fig. 8). FOT has demonstrated reasonable accuracy to detect apnea events since its early days [149].

The main limitation of event classification algorithms is that they still cannot reliably discriminate between central and obstructive *hypopneas*. Most devices, in the absence of apneas, will simply increase the pressure if breathing instability is detected, regardless of whether the flow oscillations are determined by central or obstructive causes. While one might argue that obstructive vs. central hypopnea differentiation is difficult even during manual scoring, it would be crucial for the device to accurately titrate pressure in response to obstructive events alone. Some algorithms (Löwenstein Medical Technology) combine the FOT approach with attempts at flow contour analysis (flatness vs. roundness in the flow profile; Fig. 8) to recognize different types of hypopneas [148]: this approach led to abolishing of most obstructive events, with 83% of central events not triggering a PAP increase. Yet, only 66% of manually scored events were detected by the device and, of those, just 2/3rd were of the same type as in the PSG scoring.

**Fig. 9:**
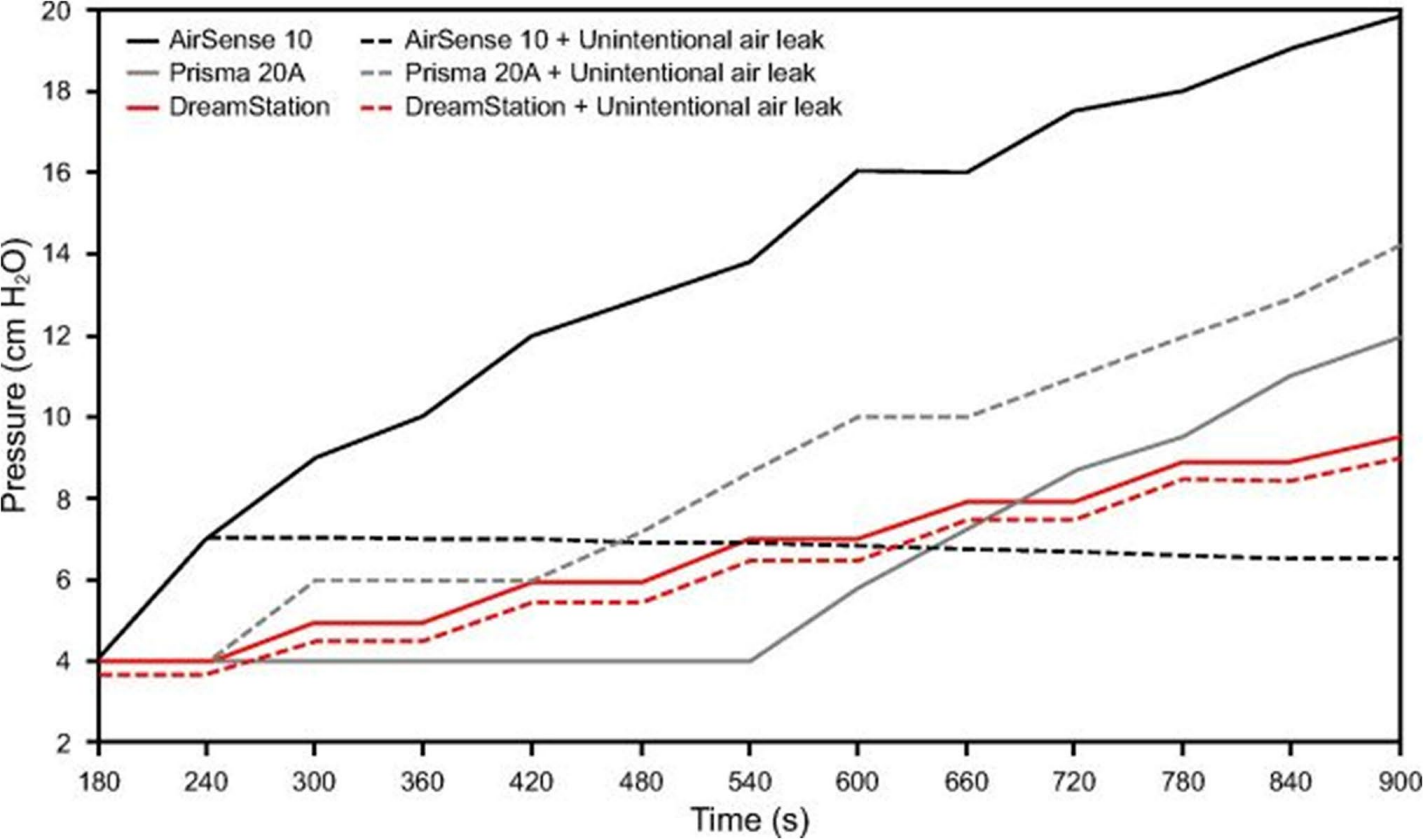
Pressure changes according to simulated obstructive apnea events with and without unintentional leak. The DreamStation device (soft algorithm) demonstrates smooth increases in pressure following onset of events, but minimal adjustments (Δ*P* = 0.5 cmH_2_O) when leak is introduced along with events. By contrast, the AirSense 10 device (aggressive algorithm) shows a significant increase in pressure with events (maximal pressurization = 19.8 cmH_2_O), but a modest response when leak is introduced (maximal pressurization = 6.5 cmH_2_O, maximal Δ*P* = 13.3 cmH_2_O, mean Δ*P* = 7.4 cmH_2_O). The Prisma 20 A device (soft algorithm) exhibits higher pressure levels with unintentional leak (Δ*P* = 10.2 cmH_2_O; a behavior possibly influenced by inaccurate estimation of unintentional leak and subsequent overcompensation) compared to without leak (Δ*P* = 8.0 cmH_2_O), with the device starting to titrate only after 7th event (⁓540th second). All these devices did not raise pressure when a central event was simulated. Overall, aggressive algorithms result in higher mean pressure biases (Δ*P*, difference of pressure with and without leak) than soft algorithms. Similar results were obtained when hypopneas were simulated. Reproduced with permission from Fasquel et al. [120]

Once the algorithm identifies the event type, one of two distinct pressure adjustment strategies, which varies by brand, follows. Broadly, these titration approaches are categorized as aggressive or soft. Aggressive algorithms react quickly with larger pressure adjustments to detected events, aiming for a rapid control of upper airway patency. However, they may increase the risk of arousals, mouth leak, over-titration, or even trigger central events. On the other hand, they generally handle leak better (Fig. 9) [120], i.e., allowing for less increase in pressure in the presence of a leak. Soft algorithms increase pressure more gradually, in theory prioritizing comfort, but they may be more prone to allowing residual events, flow limitation, and arousals. Additionally, they tend to be more accommodating with leak (Fig. 9).

In summary, event type discrimination remains a challenge for APAP and CPAP algorithms. Current technologies continue to be far from perfect in recognizing central vs. obstructive events, especially when it comes to hypopneas. Overall, algorithms incorporating FOT and those using a softer titration approach tend to perform better, but, once again, manual review of output waveforms is always recommended to ensure accurate patient management.

## Conclusions and future directions

APAP represents an important technological advancement, offering efficacy and reduced costs when compared to manual, in-laboratory titration while providing the clear advantage of saving time and clinical resources. APAP is also largely equivalent to CPAP in terms of efficacy and adherence long-term. Although not every patient is put on APAP chronically, virtually all OSA patients are now given an APAP as their first device. Indeed, the auto-adjusting and the AED algorithms streamline outpatient follow-up of OSA patients, making APAP generally a practical choice for both clinicians and patients. Thus, at least from these standpoints, APAP constituted an important technological advancement in OSA management.

However, several critical gaps remain. From a clinical-practical angle, it is still not clear whether the long-term effect of APAP on health outcomes is truly equal to that of CPAP. More research is needed on this topic, noting that existing data support fixed-level CPAP as starting therapy in patients with high blood pressure or chronic kidney disease. Second, the default 4–20 cmH_2_O APAP pressure range many patients are prescribed with should be reconsidered. As a general rule, for patients in whom APAP is the preferred treatment option, we recommend setting the pressure around the P90/P95 (e.g., ± 2 cmH_2_O P90/P95). Narrowing PAP limits or even switching to CPAP, thereby preventing excessive PAP fluctuations, becomes particularly important when high arousal index, large leak or signs of TECSA emerge. Third, the lack of algorithm transparency continues to hinder clinicians’ ability to optimize treatment, as different devices respond variably to the same patient physiology. While APAP algorithms have improved over the years, their effectiveness may be compromised by proprietary limitations that are not readily apparent to the sleep clinician. These include inconsistent event detection or inaccurate event classification. Greater transparency about algorithm coding—potentially through a shared, editable source—could not only help clinicians navigate the APAP “black box”, but also accelerate innovation through community-driven advancement. High-resolution waveforms—including night-to-night variations, leak patterns, and event flags—should also become standard outputs across all devices to allow for a more comprehensive view of treatment efficacy and residual events. In fact, most PAP devices measure and store airflow and pressure data, but only display automated charts of residual events and compliance indexes [117]. An easy way to access detailed device data should be desirable to improve upon output interpretation and patient management. Calls for improved algorithm transparency date back to at least 2006 [144], yet little has changed since. Fourth, combining other existing monitoring technologies with APAP algorithms may offer further clinical and economical advantage. For example, integrating SpO_2_ could enhance event detection without requiring major “under-the-hood” engineering changes [150]. This would refine pressure adjustments and possibly offer a means to estimate OSA endotypes [151]. Considering that oximeters are relatively inexpensive, such a simple addition could represent a major step forward in OSA treatment titration and follow-up (Fig. 5).

Finally, APAP use is recommended only for otherwise healthy OSA patients [12]. Its role in those with comorbidities remains poorly defined. Future studies should focus on the risk/benefit ratio and possible contraindications of APAP vs. in-laboratory PAP titration in populations with co-existing disease.

Addressing these knowledge gaps is essential to ensuring APAP is not just a convenient alternative to CPAP, but a truly effective and personalized therapy for all patients with OSA. Despite the availability of several APAP strategies and algorithms, most patients tend to get the same, very standardized treatment (especially within a sleep clinic, where clinicians get “accustomed” to a specific brand or device). However, in consideration of the still poor PAP adherence, it may be time, for both clinicians and engineers, to consider strategies to deliver a therapy that is really patient centered rather than defaulting to a one-size-fits-all approach.

## Data Availability

Data about the experiment with Philips-Respironics and SleepRes APAP will be made available upon reasonable request.

## Declarations

### Institution where work was performed

None, since no data collection was involved.
